# Non-intubated anesthesia in patients undergoing video-assisted thoracoscopic surgery: A systematic review and meta-analysis

**DOI:** 10.1371/journal.pone.0224737

**Published:** 2019-11-12

**Authors:** Mei-gang Yu, Ren Jing, Yi-jie Mo, Fei Lin, Xue-ke Du, Wan-yun Ge, Hui-jun Dai, Zhao-kun Hu, Sui-sui Zhang, Ling-hui Pan

**Affiliations:** 1 Department of Anesthesiology, Affiliated Tumor Hospital of Guangxi Medical University, Nanning, Guangxi, China; 2 The Laboratory of Perioperative Medicine Research Center, Affiliated Tumor Hospital of Guangxi Medical University, Nanning, Guangxi, China; 3 Department of Anesthesiology, The People’s Hospital of Guangxi Zhuang Autonomous Region, Nanning, Guangxi, China; University of Mississippi Medical Center, UNITED STATES

## Abstract

**Introduction:**

Non-intubated anesthesia (NIA) has been proposed for video-assisted thoracoscopic surgery (VATS), although how the benefit-to-risk of NIA compares to that of intubated general anesthesia (IGA) for certain types of patients remains unclear. Therefore, the aim of the present meta-analysis was to understand whether NIA or IGA may be more beneficial for patients undergoing VATS.

**Methods:**

A systematic search of Cochrane Library, Pubmed and Embase databases from 1968 to April 2019 was performed using predefined criteria. Studies comparing the effects of NIA or IGA for adult VATS patients were considered. The primary outcome measure was hospital stay. Pooled data were meta-analyzed using a random-effects model to determine the standard mean difference (SMD) with 95% confidence intervals (CI).

**Results and discussion:**

Twenty-eight studies with 2929 patients were included. The median age of participants was 56.8 years (range 21.9–76.4) and 1802 (61.5%) were male. Compared to IGA, NIA was associated with shorter hospital stay (SMD -0.57 days, 95%CI -0.78 to -0.36), lower estimated cost for hospitalization (SMD -2.83 US, 95% CI -4.33 to -1.34), shorter chest tube duration (SMD -0.32 days, 95% CI -0.47 to -0.17), and shorter postoperative fasting time (SMD, -2.76 days; 95% CI -2.98 to -2.54). NIA patients showed higher levels of total lymphocytes and natural killer cells and higher T helper/T suppressor cell ratio, but lower levels of interleukin (IL)-6, IL-8 and C-reactive protein (CRP). Moreover, NIA patients showed lower levels of fibrinogen, cortisol, procalcitonin and epinephrine.

**Conclusions:**

NIA enhances the recovery from VATS through attenuation of stress and inflammatory responses and stimulation of cellular immune function.

## Introduction

Video-assisted thoracoscopic surgery (VATS), a common diagnostic and therapeutic technology using modern video technology and high-tech equipment, has the advantages of minimal trauma and incision, reduced pain and reliable curative effect[1]. Contrary to conventional thoracotomy, VATS relies on a surveillance screen, and the operation is perform using special surgical instruments through three or four, 1.5-cm chest wall incisions[2,3]. For patients with stage Ia with tumor diameter smaller than 2 cm, VATS lobectomy can reduce the loss of lung function and improve the quality of life of patients after operation[4]. The National Comprehensive Cancer Network (NCCN) guidelines also recommend VATS lobectomy as the standard procedure for resectable lung cancer[5].

In thoracic surgery, double-lumen endobronchial intubation and pulmonary isolation technology after general anesthesia can protect the contralateral bronchus and lung tissue from contamination while fully exposing the field of operation[6]. Pulmonary sequestration using a double-lumen bronchial catheter or bronchial occlusion tube can cause such complications as throat pain, nausea, and hemoptysis[7,8]. Especially under shallow anesthesia, tracheal intubation leads to severe cough, suffocation or bronchospasm, excessive excitation of the autonomic nervous system, arrhythmias, bradycardia, ventricular premature beats, ventricular fibrillation and even cardiac arrest[9].

To reduce complications of tracheal intubation and minimize the impact of one-lung ventilation (OLV), the non-intubated (NIA) or awake VATS technique has been attempted from pleural biopsy to lobectomy[10,11]. NIA is technically feasible and safe in patients undergoing major surgery such as those undergoing lobectomy for lung cancer[12]. Whether NIA offers a good benefit-to-risk ratio for certain patient groups remains unclear[13,14], such as for patients with effective persistent hypoxemia, carbon dioxide retention, extensive pleural adhesions or severe cough. Nevertheless, NIA has the potential to improve clinical recovery and prevent stress responses, particularly in high-risk subgroups such as those with impaired pulmonary function[15–17]. In addition, VATS lung metastasectomy with NIA can trigger milder immunological and inflammatory responses than intubated general anesthesia (IGA)[18]. However, NIA application is limited by several factors, including the difficulty to control airway management and the need for a highly experienced anesthesiologist.

In view of the limitations and the controversy of NIA technique, we aimed to perform an updated systematic review and meta-analysis comparing NIA and conventional IGA for adult’s thoracic surgery in terms of mortality, other clinical and physiological outcomes, and adverse events.

## Materials and methods

This systematic review was conducted in accordance with the methodology of the Preferred Reporting Items for Systematic Reviews and Meta-Analyses (PRISMA) statement[19] from Cochrane Collaboration.

### Search strategy

Systematic methods were used to identify published randomized controlled trials or retrospective case-control studies that enrolled patients who underwent any type of VATS surgery with NIA or awake VATS in comparison with IGA. There were no restrictions on publication year or country. Only articles published in English were considered. To identify all relevant studies, we manually searched MEDLINE/Pubmed (from 1946 to April 2019), Embase/OvidSP (from 1974 to April 2019), and the Cochrane Central Register of Controlled Trials (CENTRAL) (from inception in 1965 to April 2019, see S1 File). Moreover, we manually screened the reference lists of included studies and review articles in order to identify studies that were not found in the original database search.

All studies comparing NIA and IGA in adults undergoing thoracic surgery were eligible for inclusion. All combinations of VATS were included, such as lung lobectomy, metastasectomy, segmentectomy, sympathectomy, lung volume reduction surgery; and all NIA strategies, including intercostal and vagal nerve block (INB), thoracic epidural anesthesia (TEA), TEA combined with INB and the Olympus LTF-160 semi-rigid pleuroscope) and target anesthesia.

In this review, we defined lung or pleural biopsy and pleurodesis, sympathectomy, talc pleurodesis surgery, bullectomy and wedge resection as minor thoracic surgery. Moderate thoracic surgery was defined as video-assisted flexible thoracoscopic surgery (VAFTS) decortication, lung volume reduction surgery, mediastinal tumor resection and VATS for non-oncological thoracic disease. Major thoracic surgery was defined as lobectomy, metastasectomy and segmentectomy.

### Data extraction

Three authors (Y.M.G., R.J., and M.Y.J.) used a predefined list of terms to independently identify the studies. After excluding all duplicated studies, the titles and abstracts of the potentially relevant publications were screened. If a final decision could not be made after reading the title and abstract, the full texts was assessed. Any conflicts among the three authors regarding the selection of studies was resolved through reassessment by a fourth author (F.L.). Inter-rater κ for study inclusion was 0.68, corresponding to “good” agreement[19].

For comparing patients undergoing NIA or IGA, the primary outcome was length of hospital stay. The secondary outcomes included estimated cost for hospitalization, postoperative chest tube duration, postoperative fasting time, cellular immune function, and stress and inflammatory response.

### Quality assessment

The methodological quality evaluation was performed using Review Manager (RevMan, Version 5.3. Copenhagen: The Nordic Cochrane Centre, The Cochrane Collaboration, 2014). For assessment of the risk of bias in randomized controlled trials (RCTs), two review authors (L.F. and D.X.K.) independently evaluated the methods of random sequence generation, group allocation, blinding of participants and outcome assessment, adequacy of analyses, and completeness of reporting using previously described methods[20,21]. We did not assess three domains of bias (random sequence generation, allocation and blinding) that not applicable to retrospective or observational studies[22]. Two review authors (L.F. and D.X.K.) independently assessed the methodological quality of the these included studies in terms of bias of selection, performance, detection, and attrition.

### Statistical analysis

Pooled results are shown as a summary odds ratio (OR) for dichotomous variables or pooled standard mean difference (SMD) for continuous variables, together with 95% confidence intervals (CI). These statistical estimated were determined for each parameter using the STATA/MP 13.1 Software (StataCorp LP, College Station, TX, USA).

The heterogeneity of treatment effects between studies was measured using Higgins’ inconsistency test (*I*^*2*^). *I*^*2*^ values of 25%, 50%, and 75% are typically defined as low, moderate, and high heterogeneity, respectively[23]. We used Mantel-Haenszel fixed-effect’s model for outcomes showing low heterogeneity. If heterogeneity >50%, the DerSimonian and Laird random-effects model was applied. Data on continuous variables that were reported as median with interquartile ranges were converted to mean±standard deviation using an online tool (http://www.comp.hkbu.edu.hk/~xwan/median2mean.html) according to the methods of estimated sample mean[24] and estimated standard deviation[25]. Data on continuous variables represented in box plots or histograms were digitized using PlotDigitizer Software 2.6.8 (Reversion October 27 2015, Sun Microsystems, Philippe Zeller, French).

The probability of publication bias was assessed using Begg’s test and Egger’s tests. The between-trial heterogeneity was explored by stratifying studies by the following characteristics: type of study design (RCT, retrospective study or observational study), type of surgery (minor, moderate or major), and type of NIA (INB, TEA or other anesthesia). Meta-regression was adjusted for known confounders including sample size and patient age, as well as surgery and NIA type. Results were reported as coefficients with associated 95% CIs and standard errors (SEs). The adjusted R^2^ showed the relative change in heterogeneity, with a negative value suggesting that covariates predicted less heterogeneity than expected by chance. Adjusted linear correlation trends with 95% CIs were constructed for overall data as well as for subgroup analyses. Subgroup meta-regression analyses were performed for the following subsets of patients: (1) NIA group undergoing minor surgery, (2) NIA group undergoing moderate surgery, (3) NIA group undergoing major surgery, (4) IGA group undergoing minor surgery, (5) IGA group undergoing moderate surgery and (6) IGA group undergoing major surgery.

## Results

### Search strategy

**Fig 1:**
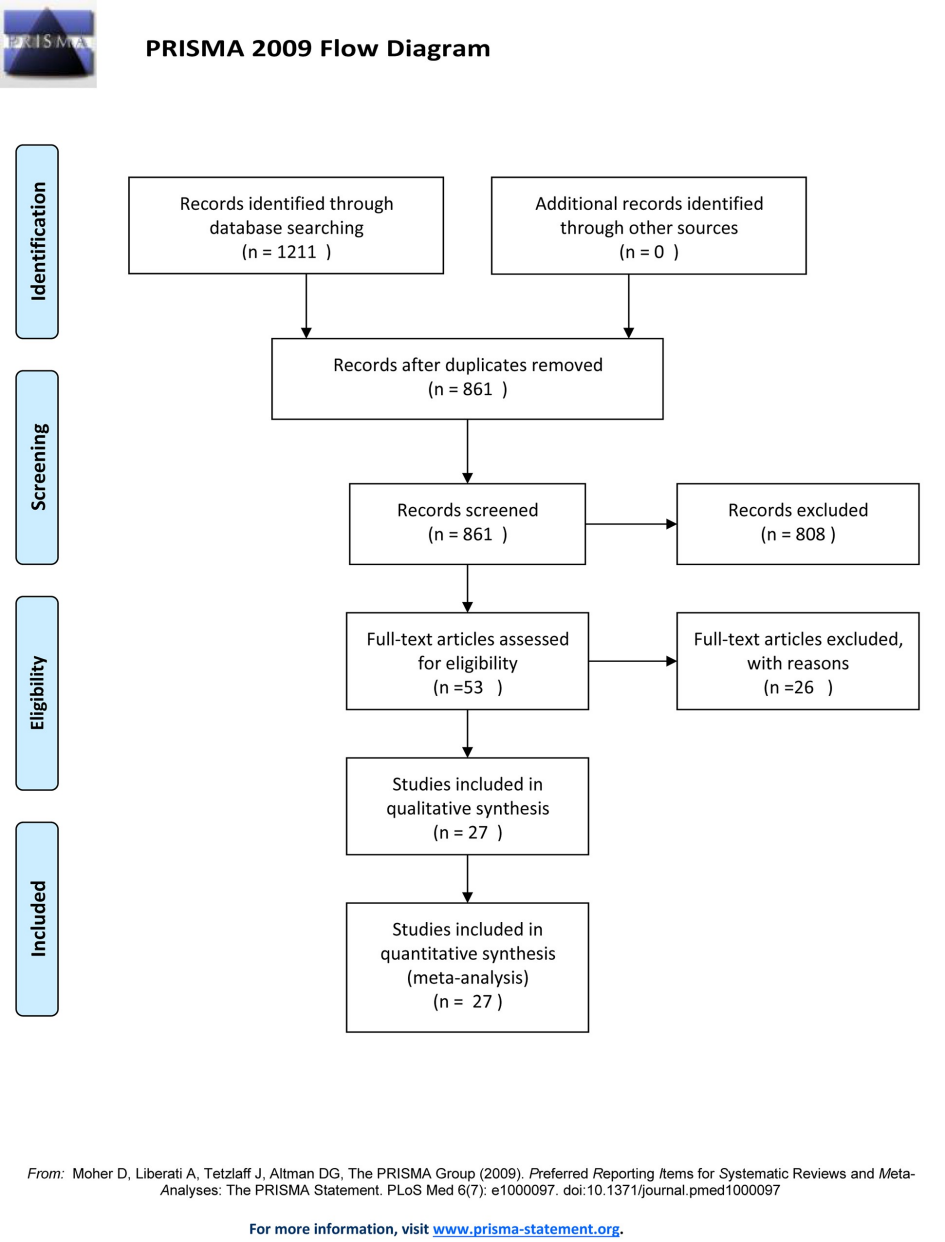
Flowchart of study selection.

The latest electronic search, conducted on 1 April 2019 in MEDLINE/PubMed, CENTRAL, and Ovid Embase databases, identified 1211 citations including 366 duplicated studies. After reviewing the title and abstract of 861 studies, 53 studies were selected for further evaluation according to our inclusion and exclusion criteria. After screening the full-texts of these 53 publications, 27 studies were selected for inclusion in the meta-analysis while 26 studies were excluded, comprising 19 studies that were not comparative but reported the clinical data for NIA VATS patients without a comparison or control group, three studies that were unpublished RCTs, and four studies were published in the supplementary information of other articles. Teams of two authors independently reviewed the titles and abstracts of each publication. The data were extracted from the 27 included studies according to our predefined inclusion and exclusion criteria, provided in S1 Table. The PRISMA flowchart (Fig 1) summarizes the process of articles search and selection.

### Characteristics of included studies

The selected 27 studies included 2929 patients. The median age of study participants was 56.8 years (range 21.9–76.4), and 1802 (61.5%) were men (Table 1). Six studies[11,26–30] were prospective RCTs published from 2004 to 2015, and four studies[12,31–33] were observational comparative studies. Seventeen studies[10,15–18,34–45] had a retrospective cohort designs. One study was designed as a retrospective, case-control study[37]. Two studies were retrospective analyses with propensity score matching[17,39]. One study was a retrospective, case-matched study using case-to-case comparison by computer according to the following clinical features: age, gender, performance status, type of previous oncologic therapy and tumor, and length of oncology history[42].

**Table 1:** Characteristics of participants and interventions.

Characteristic	NIA (*n* = 1465)	IGA (*n* = 1464)	Total (*n* = 2929)	*P* value
**Age (year)**	57(48.6–64.0)	56.6(49.7–64.4)	56.8(48.8–64.0)	0.914
**Gender (male)**	916(62.53%)	886(60.52%)	1802(61.52%)	0.264
**Surgery type**				
** MNS (*n*, %)**	771(26.32%)	812(27.72%)	1583(54.04%)	0.124
** MDS (*n*, %)**	202(6.90%)	189(6.45%)	391(13.35%)	0.485
** MJS (*n*, %)**	492(16.80%)	463(15.81%)	955(32.61%)	0.258
**Anesthesia of non-intubated surgery**				
** TEA (*n*, %)**	825(28.20%)	869(29.67%)	1694(57.87%)	0.095
** INB (*n*, %)**	443(15.12%)	377(12.87%)	820(28.00%)	0.007
** Other (*n*, %)**	197(6.69%)	218(7.44%)	415(14.13%)	0.263

Comment: Lung or pleural biopsy and pleurodesis, sympathectomy, talc pleurodesis surgery, bullectomy and wedge resection were defined as minor thoracic surgery (MNS); video-assisted flexible thoracoscopic surgery (VAFTS) decortication, lung volume reduction surgery, mediastinal tumor resection and one study reported VATS for non-oncological thoracic disease were considered as moderate thoracic surgery (MDS); lobectomy, metastasectomy and segmentectomy were classified as major thoracic surgery (MJS). VATS, video-assisted thoracoscopic surgery; TEA, thoracic epidural anesthesia; INB, intercostals nerve blockade.

Eight studies[10,26,28,29,31,35,37,43] including 672 participants compared NIA (*n* = 337) with IGA (*n* = 335) in VATS wedge resection. Five studies[12,17,26,34,39] included 646 patients with lung cancer undergoing VATS lobectomy, of which 325 participants underwent non-intubated VATS and 321 patients intubated VATS. Four studies[11,32,41,45] recruited 279 patients undergoing unilateral non-resectional lung volume reduction surgery. Three studies[37,38,40] included 248 consecutive participants undergoing elective minor VATS procedures including lung biopsy and pleural biopsy. Another three studies[18,27,30] included 552 cases who had malignant pleural effusion as well as some who had unilateral introflexing plication of the most emphysematous lung regions to schedule for VATS talc pleurodesis. Two studies[15,18] comprised 129 participants with pulmonary oligometastases to undergo VATS metastasectomy. Two studies[36,44] reported 71 participants undergoing awake video-assisted thoracoscopic pleural decortication.

A total of 1465 subjects (50.0%) underwent non-intubated or awake VATS, and another 1464 (50.0%) received intubated VATS. Nineteen studies[11,12,16,17,26–33,35,38,39,41,43–45] performed NIA under TEA alone or combined with other intravenous anesthesia, or INB. Six additional studies[10,15,18,34,36,42] administered INB combined with vagal blockade and intravenous anesthesia. In addition, one study performed paravertebral block, intercostals nerve block, or local infiltration combined with propofol (1.0–2.0 mg/kg) and sevoflurane at a minimum alveolar concentration of 0.8–1.0[37]. In another study, awake VATS was performed with an Olympus LTF-160 semi-rigid pleuroscope, and a 10 mm flexible port, and most patients were sedated with combinations of opioids, benzodiazepines or propofol[40]. All intubated anesthesia in the included studies was performed as general anesthesia with double-lumen bronchial intubation for OLV.

### Quality assessment

**Fig 2:**
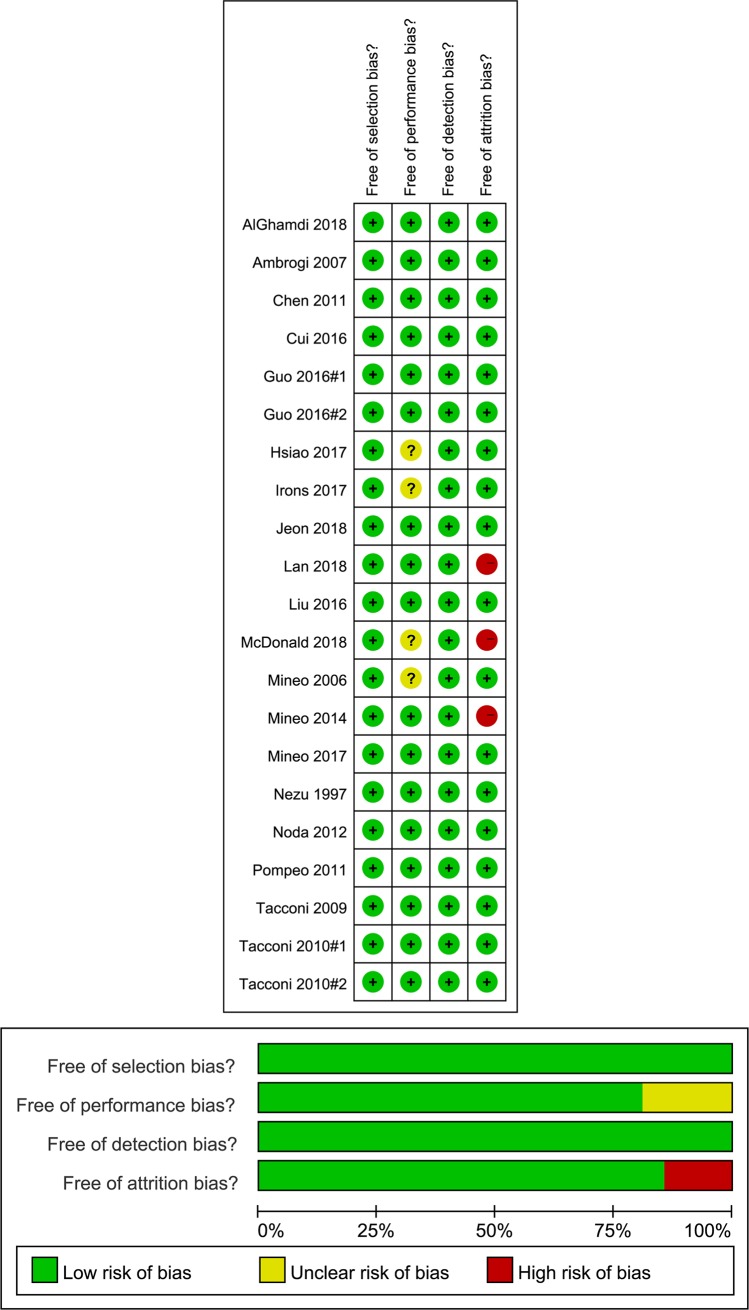
Risk of bias assessment in retrospective study or observational studies. (A) Risk of bias summary. (B) Risk of bias graph.

The risk of selection and detection bias was considered low in all retrospective or observational studies[10,12,15–18,31–45] because a consecutive sample of a clearly defined population was chosen, and medical treatment records and pathology documents were reviewed. There were 17 studies[10–12,15–18,26–35,38,39,42–45] in which the performance bias was low; in four studies[36,37,40,41], it was not specifically state. Attrition bias was moderate in 18 studies[10–12,15–18,26–38,41,43–45], and high in 3 studies[39,40,42], since several participants abandoned the study (Fig 2).

**Fig 3:**
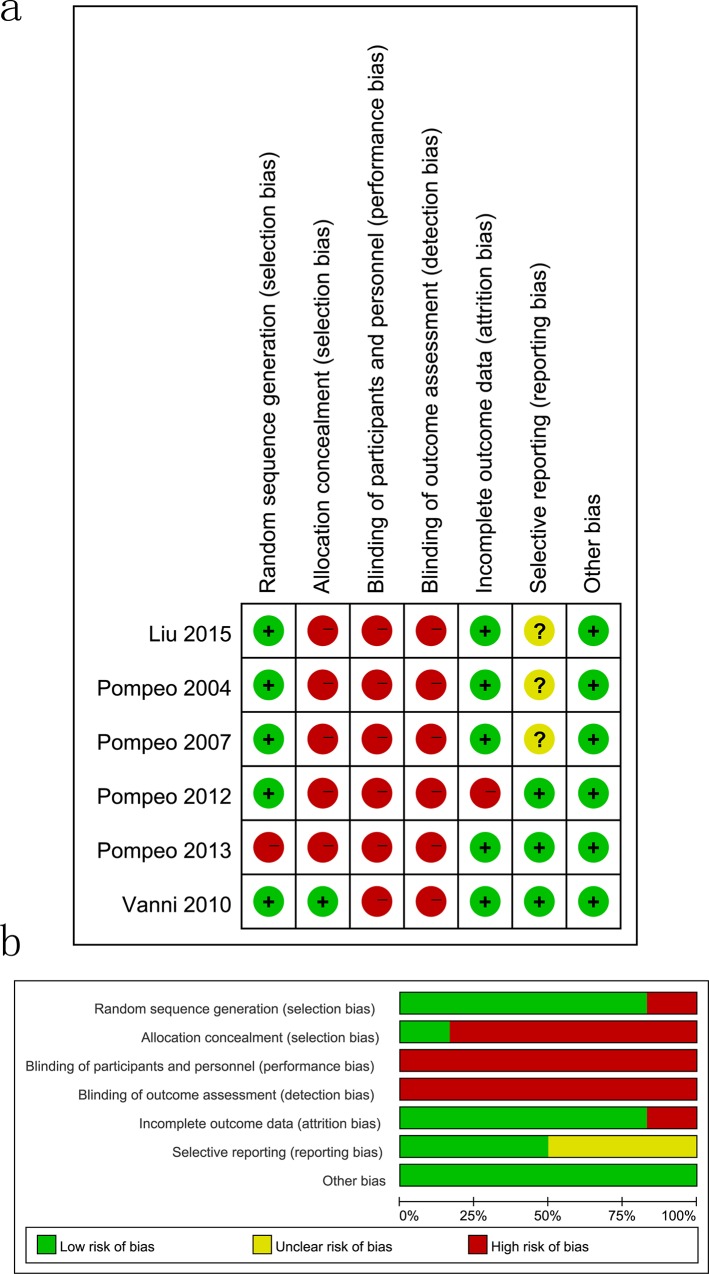
Risk of bias assessment in randomized controlled trials (RCTs). (A) Risk of bias summary. (B) Risk of bias graph.

The randomization was adequately generated in five studies[11,26,28–30], while the rest did not provide information[27]. Group allocation was inadequate in five publications[11,26–29], where surgeons were notified at the time of surgery by telephone contact with the statistical department. None of the included RCTs[11,26–30] described the blinding of participants, and the blinding of study outcomes assessment was unclear. In five studies[26–30], the potential bias due to incomplete outcome data was low, while it was high in one study where 24 patients refused randomization, while three patients required intraoperative conversion to general anesthesia (two patients in the awake group) or thoracotomy (one patient in the control group)[11]. For three studies[26,28,29] selective reporting was unclear, while another three RCTs were not registered in an official registry[11,27,30]. Finally, we judged all studies to be at low risk of other biases, as cases of NIA were followed up clinically with an objective verification of VATS[11,26–30] (Fig 3).

### Primary outcomes

**Fig 4:**
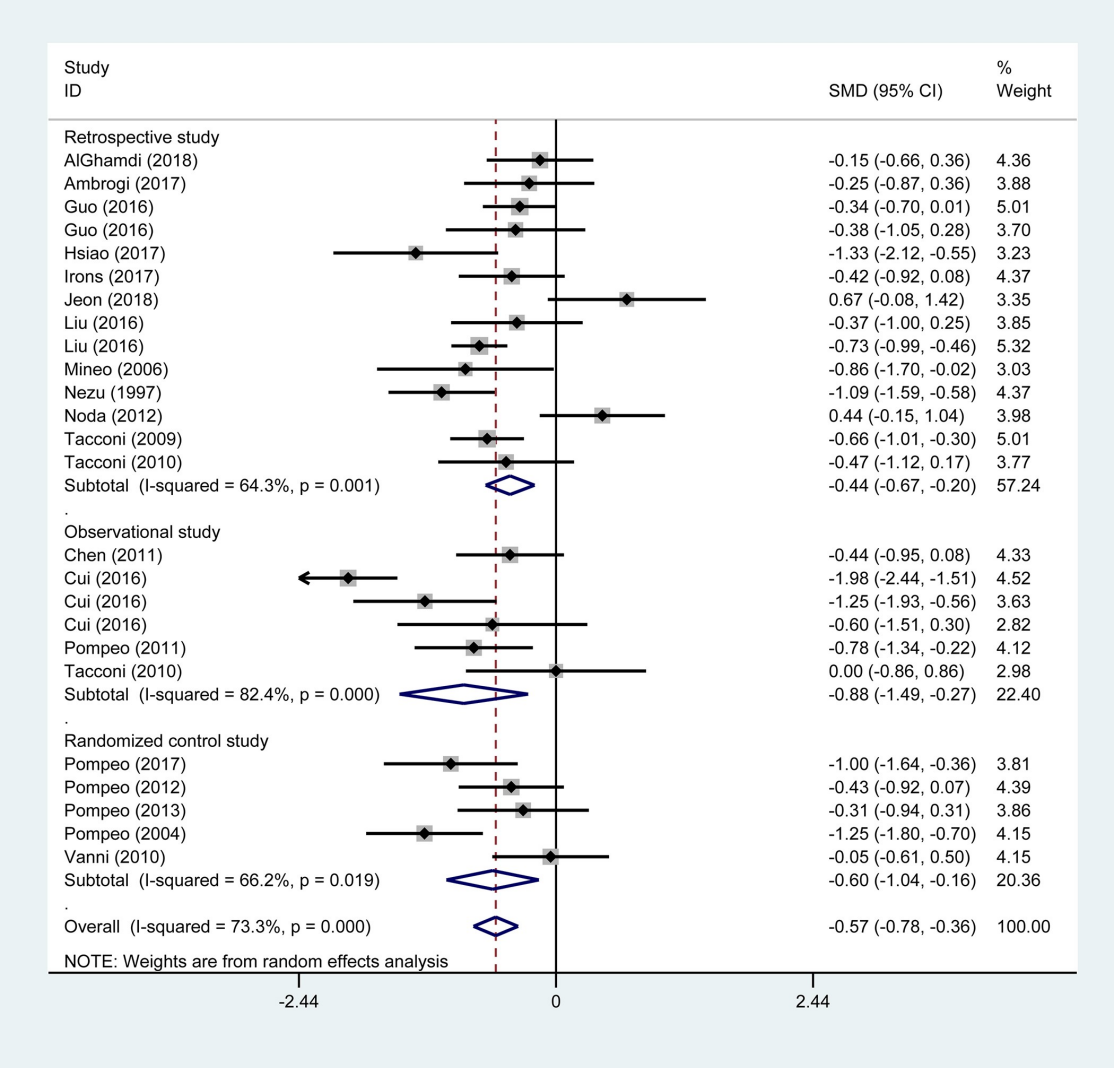
Pooled risk for hospital stays with non-intubated anesthesia versus intubated anesthesia, stratified by study design. Abbreviations: CI, confidence interval, SMD, standard mean difference.

Twenty-two studies reported the patient hospital stay after surgery[10–12,15–17,26–29,31–38,41,43–46]. In the primary analysis including these 22 studies, NIA significantly reduced postoperative hospital stay (SMD -0.57 days, 95% CI -0.78 to -0.36, *P* = 0.000; Fig 4). In the analysis stratified by study design, decreased hospital stay was found in retrospective studies (13 trials, 1018 patients; SMD -0.44 days; 95% CI -0.67 to -0.20), observational studies (four trials, 306 patients; SMD -0.88 days; 95% CI -1.49 to -0.27) and RCTs (five studies, 256 patients; SMD -0.60 days; 95% CI -1.04 to -0.16) (Fig 4). Further analysis revealed that this difference was mainly driven by a reduced days of hospital stay in patients receiving NIA in major surgery (six studies, 755 participants; SMD -0.68 days; 95% CI -1.06 to -0.30) and moderate surgery (seven studies, 371 participants; SMD -0.64 days; 95% CI -0.86 to -0.41), but similar results of hospital stay were found between NIA and IGA patients undergoing minor surgery (nine studies, 454 participants; SMD -0.40 days; 95% CI -0.82 to 0.02).

NIA patients in the subgroup of TEA (17 trials, 1297 patients; SMD -0.56 days; 95% CI -0.80 to -0.31) and INB (four trials, 221 patients; SMD -0.68 days; 95% CI -1.25 to -0.11) showed shorter postoperative hospital stay than IGA patients (Table 2). Additionally, in one study of patients undergoing bullectomy and lobectomy, hospital stay was both shorter for non-intubated patients than for intubated ones (*P* < 0.001 and *P* = 0.022); however, there were no difference in hospital stay between non-intubated or intubated patients undergoing pulmonary wedge resection.

**Table 2:** Stratified analysis of outcomes based on study design, type of surgery, and type of NIA.

Endpoint	Subgroup	No. of study	SMD	95%CI	*P* value	*I*^*2*^*(%)*	*P* heterogeneity	*P* interaction
**Hospital stay (d)**	**ROS**	13	-0.44	-0.67 to -0.20	0.000	64.3	0.001	0.000
	**OS**	4	-0.88	-1.49 to -0.27	0.005	82.4	0.000	
	**RCTs**	5	-0.60	-1.04 to -0.16	0.007	66.2	0.019	
	**MJS**	6	-0.68	-1.06 to -0.30	0.000	81.6	0.000	0.000
	**MNS**	9	-0.40	-0.82 to 0.02	0.064	78.0	0.000	
	**MDS**	7	-0.64	-0.86 to -0.41	0.000	9.6	0.356	
	**TEA**	17	-0.56	-0.80 to -0.31	0.000	75.8	0.000	0.000
	**INB**	4	-0.68	-1.25 to -0.11	0.019	72.9	0.011	
	**Other**	1	-0.42	-0.92 to 0.08	0.101	NA	NA	
**Estimated cost ($)**	**ROS**	6	-3.39	-5.44 to -1.34	0.001	98.6	0.000	0.000
	**OS**	1	-2.08	-2.74 to -1.41	0.000	NA	NA	
	**RCTs**	2	-1.55	-3.47 to 0.38	0.116	92.8	0.000	
	**MJS**	3	-2.21	-3.24 to -1.18	0.000	87.3	0.000	0.000
	**MNS**	5	-3.34	-5.98 to -0.70	0.013	98.8	0.00	
	**MDS**	1	-2.08	-2.74 to -1.41	0.000	NA	NA	
	**TEA**	5	-1.42	-2.08 to -0.77	0.000	82.7	0.000	0.000
	**INB**	3	-3.72	-6.06 to -1.38	0.002	97.5	0.000	
	**Other**	1	-7.22	-8.11 to -6.33	0.000	NA	NA	
**Chest tube duration (d)**	**ROS**	8	-0.31	-0.47 to -0.15	0.000	40.5	0.108	0.000
	**OS**	1	-0.46	-0.97 to 0.06	0.082	NA	NA	
	**MJS**	4	-0.27	-0.45 to -0.10	0.002	0.0	0.681	0.000
	**MNS**	3	-0.28	-0.62 to 0.06	0.104	0.0	0.838	
	**MDS**	1	-1.56	-2.37 to -0.75	0.000	NA	NA	
	**TEA**	5	-0.30	-0.47 to -0.13	0.001	81.4	0.005	0.001
	**INB**	3	-0.56	-1.34 to 0.21	0.154	0.0	0.957	
**Postoperative fasting time (d)**	**ROS**	2	-3.12	-3.41 to -2.83	0.000	29	0.238	0.000
	**OS**	2	-2.27	-2.61 to -1.93	0.000	32.9	0.225	
	**MJS**	3	-2.80	-3.47 to -2.13	0.000	82.3	0.001	0.000
	**MNS**	1	-2.54	-2.99 to -2.10	0.000	0.0	0.460	
	**MDS**	1	-2.01	-3.11 to -0.91	0.000	NA	NA	
	**TEA**	4	-2.67	-3.12 to -2.22	0.000	71.4	0.000	0.000

Comments: NIA, nonintubated anesthesia; SMD, standard mean difference; 95%CI, 95% confidence interval; ROS, retrospective study; OS, observational study; RCT, randomized controlled trial; MJS, major surgery; MNS, minor surgery; MDS, moderate surgery; TEA, thoracic epidural anesthesia; INB, intercostals nerve blockade; NA, not applicable.

**Fig 5:**
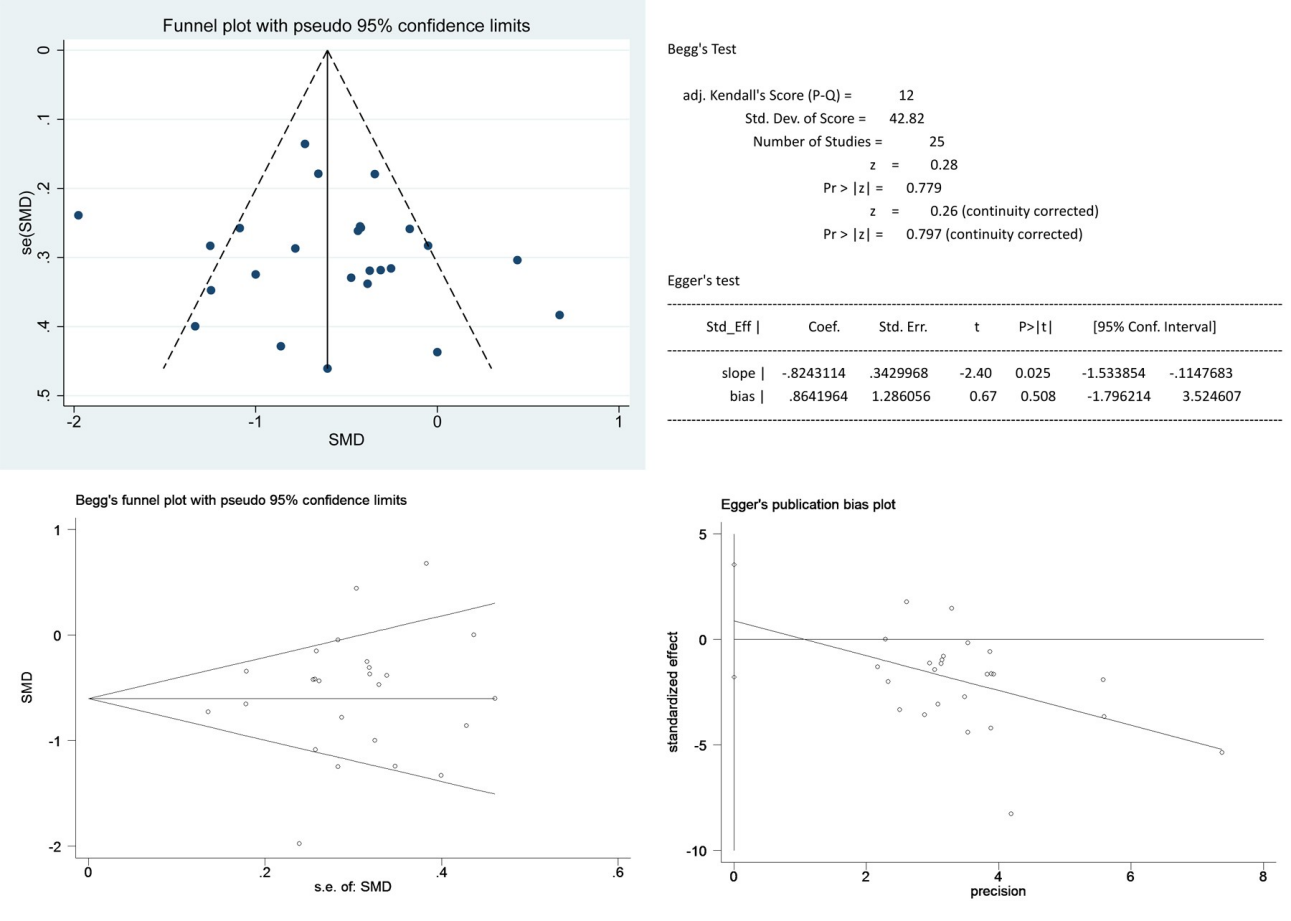
Publication bias based on data for hospital stay. (A) Funnel plot; (B) Data of Begg’s and Egger’s test; (C) Begg’s funnel plot; (D) Egger’s publication bias plot. Abbreviations: SMD, standard mean difference.

There was no evidence of publication bias by Begg’s rank correlation test (*P* = 0.779) or Egger’s rank test (*P* = 0.508) (Fig 5).

### Secondary outcomes

#### Estimated costs

**Fig 6:**
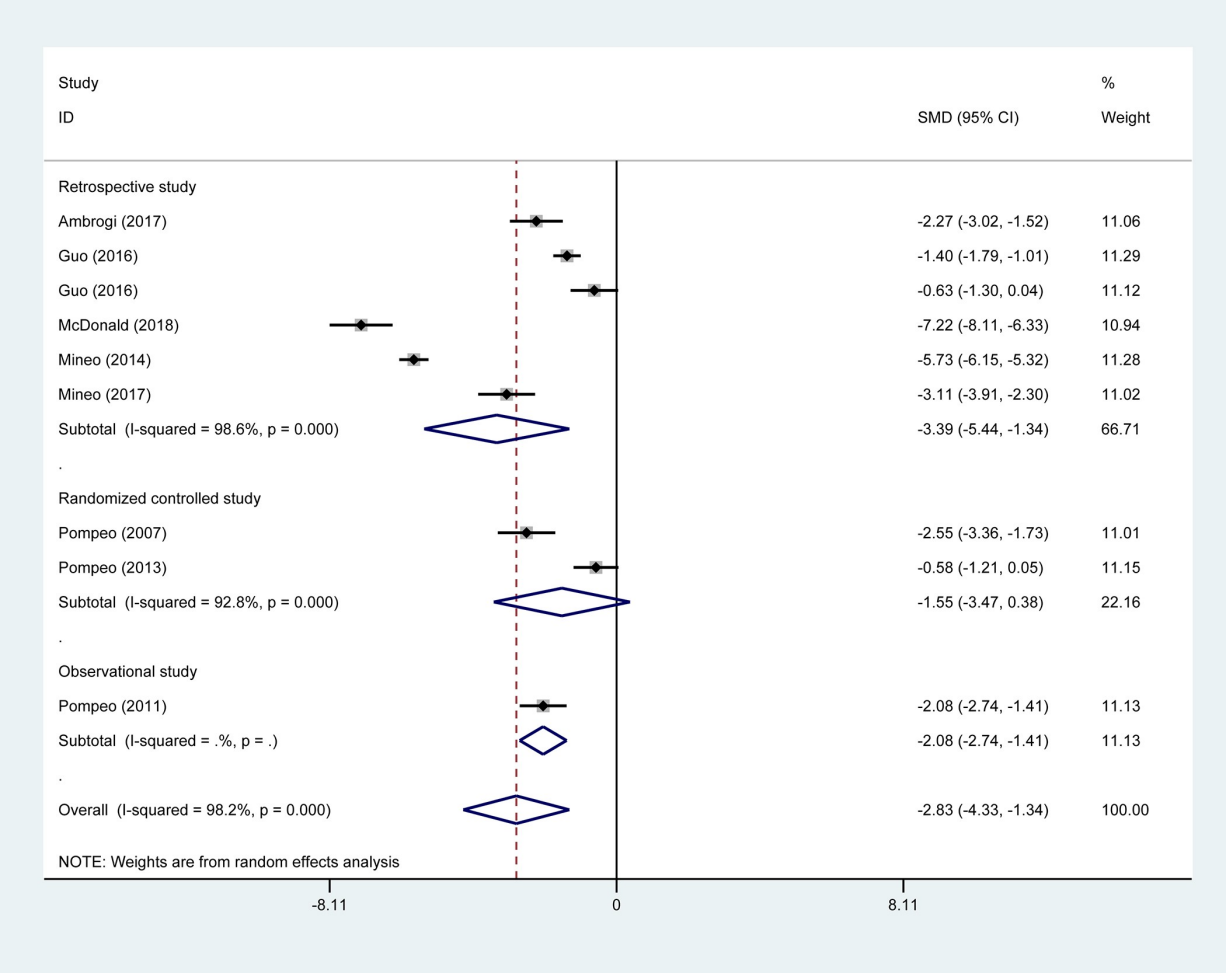
Pooled risk for estimated cost of non-intubated anesthesia versus intubated anesthesia, stratified by study design. Abbreviations: CI, confidence interval, SMD, standard mean difference.

A decrease in the estimated cost was found in favor of NIA compared with intubated VATS (SMD -2.83 US $, 95% CI -4.33 to -1.34; *P* < 0.001; nine studies with 1056 participants) (Fig 6). This finding was consistent in retrospective studies (six studies including 913 patients; SMD -3.39 US $; 95% CI -5.44 to -1.34) and in one observational study (60 patients; SMD -2.08 US $, 95% CI -2.74 to -1.41). No statistical significance difference was observed in two RCTs with 83 participants. In the analysis stratified by type of surgery, the estimated cost to VATS patients was significantly lower for NIA than for IGA participants (Table 2).

#### Chest tube duration

**Fig 7:**
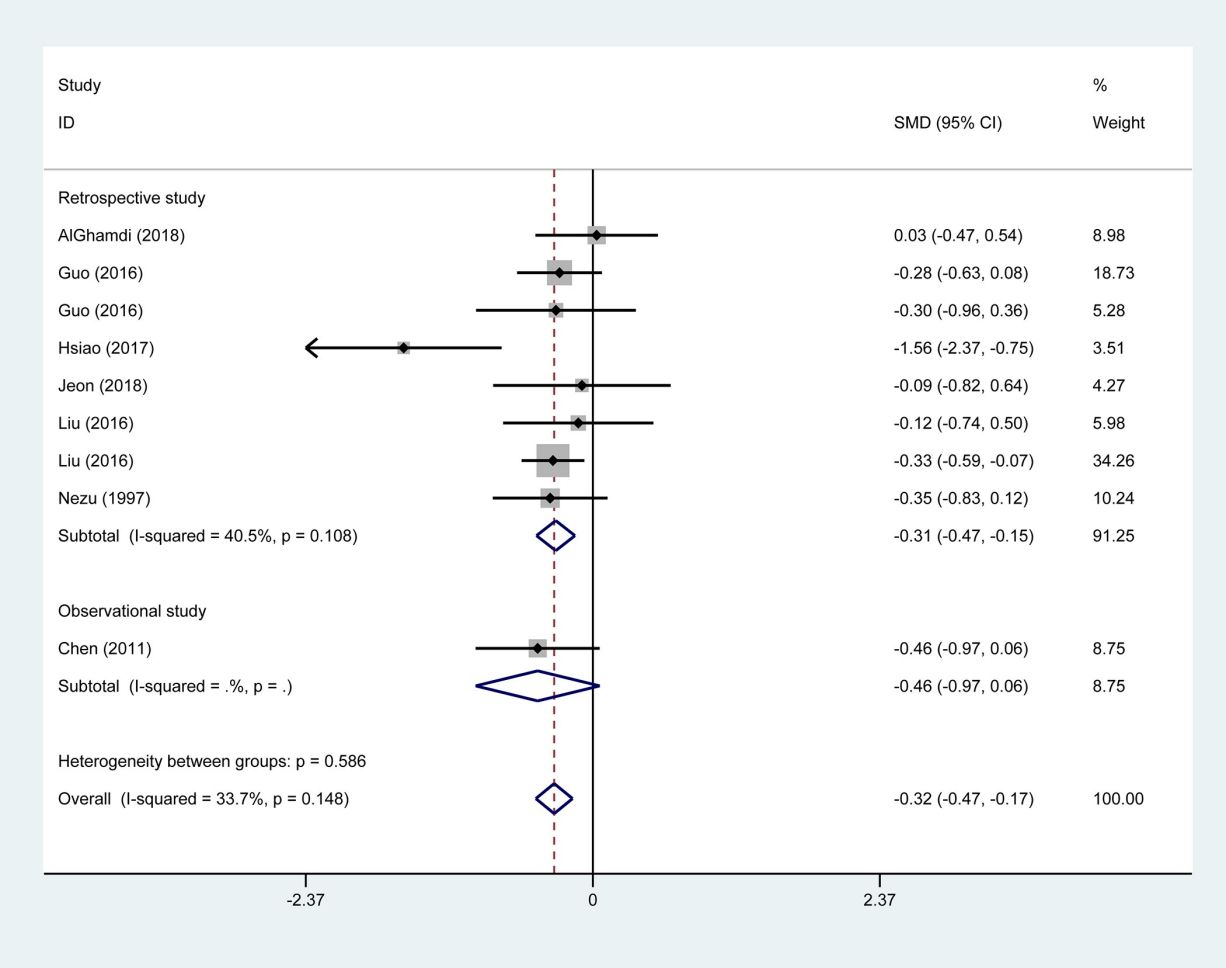
Pooled risk for chest tube duration of non-intubated anesthesia versus intubated anesthesia, stratified by study design. Abbreviations: CI, confidence interval, SMD, standard mean difference.

NIA resulted in a shortened chest tube duration after surgery compared with IGA administration (SMD -0.32 days, 95% CI -0.47 to -0.17; *P* < 0.001; eight studies with 707 participants) (Fig 7). The difference was significant in the subgroup of clinical trials (seven trials including 647 patients; SMD -0.31 days; 95% CI -0.47 to -0.15), in studies involving major surgery (four studies with 532 patients; SMD -0.27 days; 95% CI -0.45 to -0.10) and one study involving moderate surgery (33 patients; SMD -1.56 days; 95% CI -2.37 to -0.75). However, one observational study showed no significant difference (60 patients; SMD -0.46 days; 95% CI -0.97 to 0.06). Studies involving minor surgery showed comparable chest tube duration between NIA and IGA patients (three studies with 142 patients; SMD -0.28 days; 95% CI, -0.62 to 0.06). Studies involving TEA-NIA also showed shorter chest tube duration for NIA than IGA (five studies with 544 patients; SMD -0.30 days; 95% CI -0.47 to -0.13) but this was not the case for INB (three studies including 163 patients; SMD -0.56 days; 95% CI -1.34 to 0.21) (Table 2).

#### Postoperative fasting time

**Fig 8:**
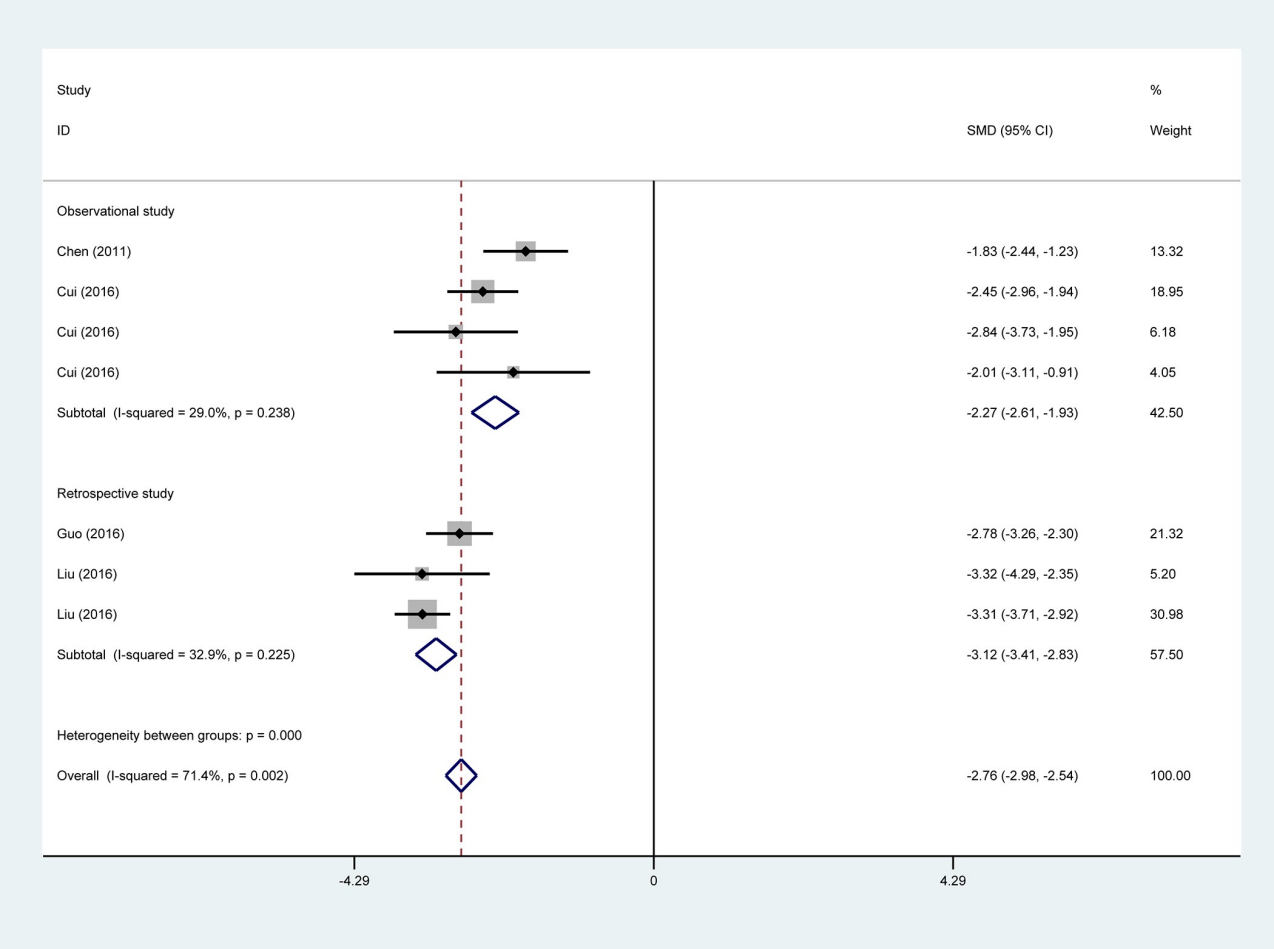
Pooled risk for postoperative fasting time of non-intubated anesthesia versus intubated anesthesia, stratified by study design. Abbreviations: CI, confidence interval, SMD, standard mean difference.

Four studies reported on postoperative fasting time[12,16,17,31]. Pooled results proved that postoperative fasting time in patients undergoing NIA VATS was shorter than in IGA patients (four studies with 637 patients; SMD, -2.76 days; 95% CI -2.98 to -2.54) (Fig 8). Subgroup analysis in study design, type of surgery and NIA calculating SMD showed an identical trend toward shorter postoperative fasting time in the NIA group than in the IGA group (Table 2).

#### Cellular immune function

We analyzed some variables to assess the effect of NIA on perioperative cellular immune function of thoracic patients. Interestingly, compared to IGA, NIA was associated with higher number of total lymphocytes (two studies with 118 patients; SMD, 0.32; 95% CI 0.08 to 0.56), T helper/T suppressor cell ratio (two studies, 118 patients; SMD 0.28; 95% CI 0.04 to 0.52) and number of natural killer (NK) cells (three studies with 176 patients; SMD 0.74; 95% CI 0.54 to 0.95). There were no significant differences in the levels of B lymphocytes, T lymphocytes, CD4+ T lymphocytes or CD8+ T lymphocytes between NIA and IGA groups (Table 3).

**Table 3:** Summary of immune function and stress response in included studies of NIA compared with IGA.

Outcome variable	No. of study	No. of patients		SMD	95%CI	*P* value	*I*^*2*^*(%)*
		NIA	IGA				
**Cellular immune function:**							
**Total lymphocytes (%)**	2	80	38	0.32	0.08 to 0.56	0.009	0.0
**T lymphocytes (%)**	2	80	38	-0.16	-0.51 to 0.19	0.369	53.5
**B lymphocytes (%)**	2	80	38	0.12	-0.12 to 0.35	0.329	0.0
**CD4+ T lymphocytes (%)**	1	25	25	-0.01	-0.33 to 0.31	0.946	0.0
**CD8+ T lymphocytes (%)**	1	25	25	-0.20	-0.53 to 0.12	0.211	0.0
**T helper/T suppressor ratio**	2	80	38	0.28	0.04 to 0.52	0.021	0.0
**NK cells (%)**	3	125	51	0.74	0.54 to 0.95	0.000	0.0
**Stress and inflammatory response:**							
White blood cell (*10^**9**^**/L)**	3	257	283	-0.68	-1.04 to -0.32	0.000	87.1
**IL-6 (pg/ml)**	3	331	257	-1.01	-1.25 to -0.78	0.000	71.9
**IL-8 (pg/ml)**	1	231	231	-0.84	-1.55 to -0.12	0.021	97.6
**IL-10 (pg/ml)**	2	100	26	0.18	-0.07 to 0.43	0.161	44.0
**CRP (mg/l)**	3	257	283	-0.66	-0.94 to -0.38	0.000	77.1
**Fibrinogen (ng/dl)**	2	242	241	-0.50	-0.61 to -0.40	0.000	41.3
**Cortisol (μg/dl)**	1	11	10	-1.85	-3.29 to -0.41	0.012	81.4
**ACTH (pg/dl)**	1	11	10	-0.75	-1.58 to 0.08	0.076	60.1
**Procalcitonin (ng/dl)**	1	231	231	-1.00	-1.39 to -0.60	0.000	92.0
**Epinephrine (ng/l)**	1	11	10	-1.17	-1.72 to -0.62	0.000	48.3
**Norepinephrine (ng/l)**	1	11	10	-0.13	-0.63 to 0.37	0.603	0.0

Comments: NIA, nonintubated anesthesia; IGA, intubation general anesthesia; SMD, standard mean difference; 95%CI, 95% confidence interval; TEA, thoracic epidural anesthesia; INB, intercostals nerve blockade; NK, natural killer; IL, interleukin; CRP, C-reactive protein; ACTH, adrenocorti cotrophic hormone.

#### Stress and inflammatory response

Compared with IGA group, NIA was associated with lower number of white blood cells (three studies with 540 patients; SMD -0.67*10^9^/L; 95% CI -1.04 to -0.32), interleukin (IL)-6 (three studies with 588 patients; SMD -1.01 pg/mL; 95% CI -1.25 to -0.78), IL-8 (one study with 462 patients; SMD -0.84 pg/mL; 95% CI -1.55 to -0.12) and C-reactive protein (CRP) (three studies with 540 patients; SMD -0.66 mg/L; 95% CI -0.94 to -0.38). However, levels of IL-10 were similar between these two groups (Table 3).

Regarding the stress responses, compared to intubated participants, NIA patients showed lower levels of fibrinogen (two studies with 483 patients; SMD -0.50 ng/dL; 95% CI -0.61 to -0.40), cortisol (one study with 21 patients; SMD -1.85 μg/dL; 95% CI -3.29 to -0.41), procalcitonin (one study with 462 patients; SMD -1.00 ng/dL; 95% CI -1.39 to -0.60), and epinephrine (one study with 21 patients; SMD -1.17 ng/L; 95% CI -1.72 to -0.62).There were no significant differences in the levels of adrenocorticotropic hormone and norepinephrine (Table 3).

### Meta-regression

**Fig 9:**
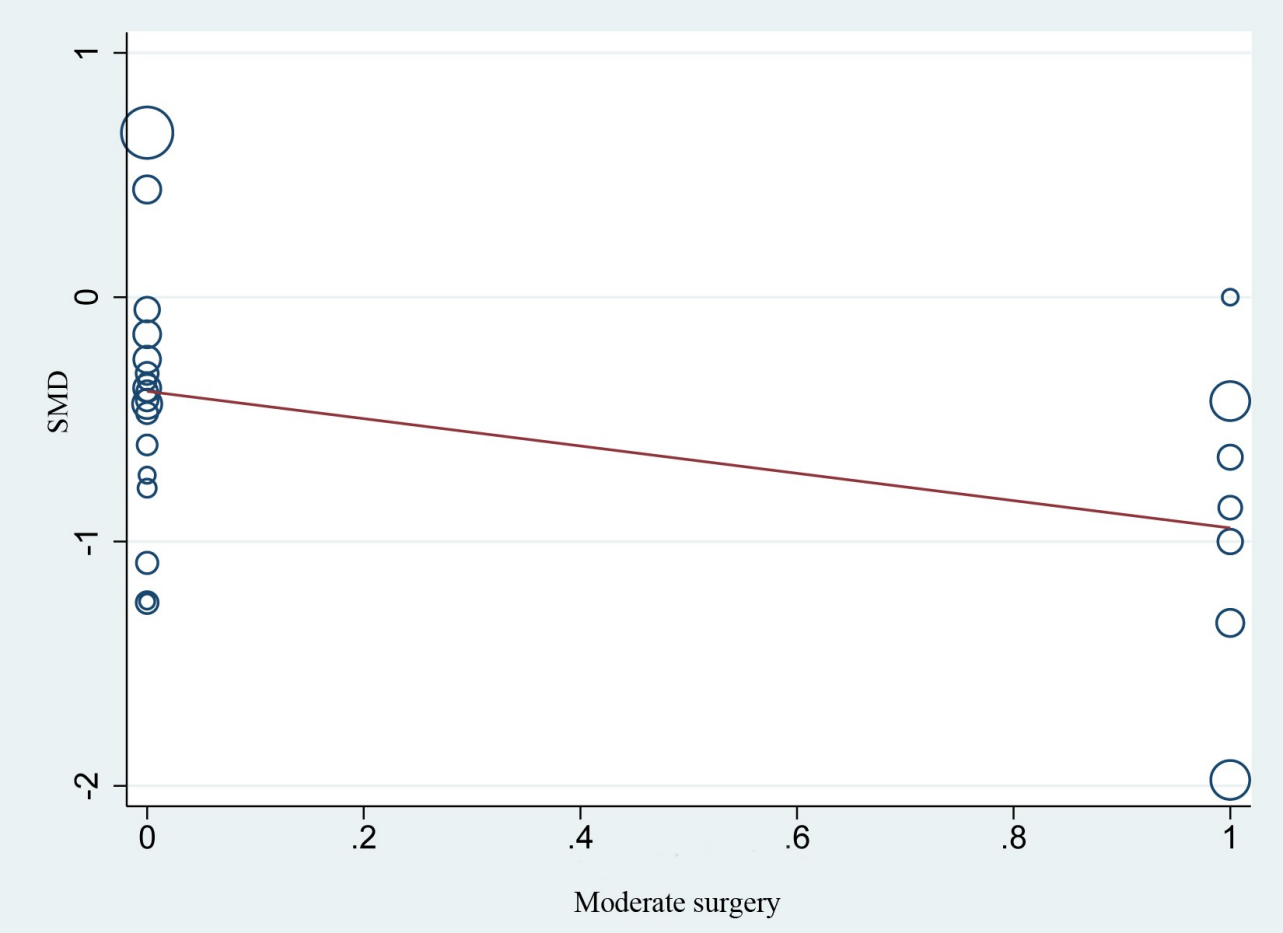
Graphic depiction of the linear correlation between moderate surgery (x axis) and the SMD of hospital stay (y axis). The solid line represents the point estimates of association between moderate surgery and the SMD of hospital stay. Bubble size is inversely proportional to the SMD of the hospital stay reported by each study (slope = -0.56%, *P* = 0.036). Abbreviations: SMD, standard mean difference.

Meta-regression analysis showed that SMD of hospital stay did not vary significantly with publication year, age, sample size or study type (all *P* > 0.05). Regarding the type of NIA, Fig 9 shows the plot of log SMD on those patients undergoing moderate surgery. The meta-regression model showed a regression coefficient b of 0.572 and a *P* value of 0.036 (see Fig 9). It suggested that the SMD for patients undergoing moderate surgery accounted for 68.0% source of inter-study heterogeneity, and removing such patients from the analysis reduced inter-study tau2 from 0.7330 to 0.2342. At the same time, meta-regression analysis confirmed that the SMD for patients undergoing minor or major surgery were not a source of inter-study heterogeneity (both *P* > 0.05). Meta-regression analyses of multiple covariates showed that SMD of hospital stay did not vary significantly with (1) type of study or type of NIA; (2) type of study and surgery, or (3) type of study, NIA and surgery (all *P* > 0.05).

## Discussion

On the basis of pooled data from 27 studies that included nearly 2929 patients undergoing VATS with NIA or IGA, we found that NIA might enhance recovery through attenuation of stress and inflammatory responses and stimulation of the cellular immune function, resulting in decreased hospital stay and estimated cost of hospitalization.

OLV and general anesthesia play a pivotal role in the activation and release of stress hormones. A previous study showed that OLV induction immediately increases cortisol and epinephrine levels while simultaneously reducing NK cells activity, suggesting that OLV significantly affects metabolism and antimicrobial immune responses[47]. Since cortisol release is regulated by circulating cytokines such as IL-6 and IL-8 as well as TNF-α, OLV may upregulate cortisol plasma level by increasing production of proinflammatory factors, with the subsequent release of these soluble inflammatory mediators into the blood circulation[31,48,49]. Sympathetic excitation and increased secretion of pituitary proadrenal cortex with upregulates of epinephrine, norepinephrine and cortisol concentrations This results in elevation of mean artery pressure heart rate and blood sugar during OLV and general anesthesia, which can be interpreted as a protective response to anesthesia-related hypoxia and hypotension. Intubation-induced stress responses should not occur in NIA, while OLV and TEA can block the afferent and efferent neural pathways to inhibit the sympathetic system[50,51]. In the present study, NIA patients showed lower levels of fibrinogen, cortisol, procalcitonin and epinephrine than IGA participants, suggesting that NIA attenuates the activation and release of stress hormones.

Surgical separation, stress response, pulmonary ischemia-reperfusion and anesthetics during VATS can activate immune inflammation, such as the release of inflammatory factors and the impairment of pulmonary function after operation[52]. Compared with the IGA group, NIA patients showed significantly lower levels of white blood cells, IL-6, IL-8 and CRP whileas levels of IL-10 were similar between the two groups. This may indicate that NIA patients experienced significantly less acute infection or tissues injury and lower levels of pro-inflammatory cytokines than intubated patients under general anesthesia. Epidural anesthesia can protect against lymphocyte suppression at 72 h postoperatively, while transiently reducing NK activity through decreased epinephrine concentration[53–55]. An epidural catheter is not administered for NIA during VATS but it can provide effective analgesia after surgery. Here, the overall level of total lymphocytes, NK cells and T helper/T suppressor cell ratio were higher in the NIA group, while levels of T lymphocytes, B lymphocytes, CD4+ T lymphocytes and CD8+ T lymphocyteswere similar between NIA and IGA patients. This suggests that NIA can ameliorate immune suppression after surgery in some aspects.

On the other hand, some anesthesiologists have proposed that NIA increases the risk of hypoxemia, hypercapnia, mediastinal oscillation and cough reflex. Therefore, anesthesiologists should always be alert to changes in vital signs during the operation. In some cases, laryngeal mask ventilation, stellate ganglion or vagus nerve block, and preoperative inhalation of lidocaine are needed, increasing anesthesiologist’s workload. In fact, NIA had proved to be a feasible and reliable technique to guarantee the stability of circulation and respiration during VATS Our results demonstrate that NIA patients realized short-term outcomes earlier than IGA patients, including shorter chest tube duration and postoperative fasting time. This accelerated recovery may help explain the lower overall estimated costs of hospitalization.

In a previous meta-analysis of patients undergoing lung resection surgery, NIA in thoracoscopic surgery favored a shorter length of hospital stay compared with IGA anesthesia, but the incidence of postoperative pulmonary complications was comparable between the two groups[13]. However, that reviews lacked a systematic search strategy, did not include an assessment of absolute changes in clinical outcomes at stress response, inflammation and cellular immune function, and did not examine perioperative outcomes. Compared with another published meta-analysis of 1684 cases[56], our study included more studies and individuals, defined hospital stay as primary outcome and provided the pooled results of estimated costs, postoperative fasting time, cellular immune function, stress and inflammatory response.

This study presents several limitations. First, the present study was not registered as a systemic review and meta-analysis in PROSPERO before it was performed. Second, the results were analyzed based on study design, type of surgery and NIA level data, but not on patient level data. Third, definitions of clinical outcomes were based on the definitions in the corresponding original studies and may therefore lack uniformity across all the studies. Fourth, pooled analysis of immune function and stress responses used data from only 1–2 studies. Fifth, the majority of the included studies were retrospective and some had small samples.

In conclusion, pooled data from nearly 2929 patients receiving VATS suggest that NIA is associated with faster, better recovery from surgery than IGA, mainly reflected in shorter hospital stay and lower estimated cost. The improvement in NIA patients was evident in those undergoing moderate or major surgery, but not in those undergoing minor surgery. This improvement was associated with attenuation of stress and inflammatory response, reduced inhibition of cellular immune function. Therefore, NIA might be a safe and feasible anesthetic strategy for VATS.

## Supporting information

S1 File Detailed search strategy for MEDLINE/Pubmed, Embase/OvidSP and the Cochrane Central Register of Controlled Trials (CENTRAL). (DOCX)

S2 File PRISMA checklist. Completed checklist of PRSIMA guidelines.
(DOC)

S1 Table Characteristics of included studies *[ordered by study ID]*. (DOC)

## References

[1] Reichert M, Kerber S, Alkoudmani I, Bodner J (2016) Management of a solitary pulmonary arteriovenous malformation by video-assisted thoracoscopic surgery and anatomic lingula resection: video and review. Surg Endosc 30: 1667–1669. 10.1007/s00464-015-4337-0 26156615

[2] Zahid I, Nagendran M, Routledge T, Scarci M (2011) Comparison of video-assisted thoracoscopic surgery and open surgery in the management of primary empyema. Curr Opin Pulm Med 17: 255–259. 10.1097/MCP.0b013e3283473ffe 21519265

[3] Chou YP, Lin HL, Wu TC (2015) Video-assisted thoracoscopic surgery for retained hemothorax in blunt chest trauma. Curr Opin Pulm Med 21: 393–398. 10.1097/MCP.0000000000000173 25978625 PMC5633323

[4] Bendixen M, Jorgensen OD, Kronborg C, Andersen C, Licht PB (2016) Postoperative pain and quality of life after lobectomy via video-assisted thoracoscopic surgery or anterolateral thoracotomy for early stage lung cancer: a randomised controlled trial. Lancet Oncol 17: 836–844. 10.1016/S1470-2045(16)00173-X 27160473

[5] Ettinger DS, Aisner DL, Wood DE, Akerley W, Bauman J, et al (2018) NCCN Guidelines Insights: Non-Small Cell Lung Cancer, Version 5.2018. J Natl Compr Canc Netw 16: 807–821. 10.6004/jnccn.2018.0062 30006423

[6] Kiss G, Claret A, Desbordes J, Porte H (2014) Thoracic epidural anaesthesia for awake thoracic surgery in severely dyspnoeic patients excluded from general anaesthesia. Interact Cardiovasc Thorac Surg 19: 816–823. 10.1093/icvts/ivu230 25035439

[7] Bahk JH (2002) Guidelines for determining the appropriateness of double-lumen endobronchial tube size. Anesth Analg 95: 501. 10.1097/00000539-200208000-00063 12145088

[8] Xue FS, Sun C, Liu GP (2017) Assessing influence of thermal softened double-lumen endobronchial tube on postoperative airway injury and morbidity: a call for methodology clarification. Br J Anaesth 118: 139–140. 10.1093/bja/aew416 28039251

[9] Innes ME (2018) First-attempt success of emergency intubation with bougie was higher than with endotracheal tube plus stylet. Ann Intern Med 169: JC40 10.7326/ACPJC-2018-169-8-040 30326084

[10] Nezu K, Kushibe K, Tojo T, Takahama M, Kitamura S (1997) Thoracoscopic wedge resection of blebs under local anesthesia with sedation for treatment of a spontaneous pneumothorax. Chest 111: 230–235. 10.1378/chest.111.1.230 8996022

[11] Pompeo E, Rogliani P, Tacconi F, Dauri M, Saltini C, et al (2012) Randomized comparison of awake nonresectional versus nonawake resectional lung volume reduction surgery. J Thorac Cardiovasc Surg 143: 47–54, 54.e41. 10.1016/j.jtcvs.2011.09.050 22056369

[12] Chen JS, Cheng YJ, Hung MH, Tseng YD, Chen KC, et al (2011) Nonintubated thoracoscopic lobectomy for lung cancer. Ann Surg 254: 1038–1043. 10.1097/SLA.0b013e31822ed19b 21869676

[13] Shi Y, Yu H, Huang L, Wang S, Chi D, et al (2018) Postoperative pulmonary complications and hospital stay after lung resection surgery: A meta-analysis comparing nonintubated and intubated anesthesia. Medicine (Baltimore) 97: e10596. 29794734 10.1097/MD.0000000000010596 PMC6392661

[14] Zheng H, Hu XF, Jiang GN, Ding JA, Zhu YM (2017) Nonintubated-Awake Anesthesia for Uniportal Video-Assisted Thoracic Surgery Procedures. Thorac Surg Clin 27: 399–406. 10.1016/j.thorsurg.2017.06.008 28962712

[15] Ambrogi V, Sellitri F, Perroni G, Schillaci O, Mineo TC (2017) Uniportal video-assisted thoracic surgery colorectal lung metastasectomy in non-intubated anesthesia. J Thorac Dis 9: 254–261. 10.21037/jtd.2017.02.40 28275472 PMC5334099

[16] Guo Z, Yin W, Pan H, Zhang X, Xu X, et al (2016) Video-assisted thoracoscopic surgery segmentectomy by nonintubated or intubated anesthesia: A comparative analysis of short-term outcome. Journal of Thoracic Disease 8: 359–368. 10.21037/jtd.2016.02.50 27076930 PMC4805789

[17] Liu J, Cui F, Pompeo E, Gonzalez-Rivas D, Chen H, et al (2016) The impact of non-intubated versus intubated anaesthesia on early outcomes of video-assisted thoracoscopic anatomical resection in non-small-cell lung cancer: a propensity score matching analysis. Eur J Cardiothorac Surg 50: 920–925. 10.1093/ejcts/ezw160 27165771

[18] Mineo TC, Sellitri F, Vanni G, Gallina FT, Ambrogi V (2017) Immunological and Inflammatory Impact of Non-Intubated Lung Metastasectomy. Int J Mol Sci 18. 10.3390/ijms18071466 PMC5535957 28686211

[19] Moher D, Liberati A, Tetzlaff J, Altman DG, Group P (2009) Preferred reporting items for systematic reviews and meta-analyses: the PRISMA statement. BMJ 339: b2535 10.1136/bmj.b2535 19622551 PMC2714657

[20] Di Nisio M, Peinemann F, Porreca E, Rutjes AW (2015) Primary prophylaxis for venous thromboembolism in patients undergoing cardiac or thoracic surgery. Cochrane Database Syst Rev: CD009658 10.1002/14651858.CD009658.pub2 26091835 PMC11024391

[21] Zheng SL, Roddick AJ (2019) Association of Aspirin Use for Primary Prevention With Cardiovascular Events and Bleeding Events: A Systematic Review and Meta-analysis. JAMA 321: 277–287. 10.1001/jama.2018.20578 30667501 PMC6439678

[22] Carbine NE, Lostumbo L, Wallace J, Ko H (2018) Risk-reducing mastectomy for the prevention of primary breast cancer. Cochrane Database Syst Rev 4: CD002748 10.1002/14651858.CD002748.pub4 29620792 PMC6494635

[23] Rucker G, Schwarzer G, Carpenter JR, Schumacher M (2008) Undue reliance on I(2) in assessing heterogeneity may mislead. BMC Med Res Methodol 8: 79 10.1186/1471-2288-8-79 19036172 PMC2648991

[24] Luo D, Wan X, Liu J, Tong T (2018) Optimally estimating the sample mean from the sample size, median, mid-range, and/or mid-quartile range. Stat Methods Med Res 27: 1785–1805. 10.1177/0962280216669183 27683581

[25] Wan X, Wang W, Liu J, Tong T (2014) Estimating the sample mean and standard deviation from the sample size, median, range and/or interquartile range. BMC Med Res Methodol 14: 135 10.1186/1471-2288-14-135 25524443 PMC4383202

[26] Liu J, Cui F, Li S, Chen H, Shao W, et al (2015) Nonintubated video-assisted thoracoscopic surgery under epidural anesthesia compared with conventional anesthetic option: a randomized control study. Surg Innov 22: 123–130. 10.1177/1553350614531662 24821259

[27] Pompeo E, Dauri M (2013) Is there any benefit in using awake anesthesia with thoracic epidural in thoracoscopic talc pleurodesis? J Thorac Cardiovasc Surg 146: 495–497.e491. 10.1016/j.jtcvs.2013.03.038 23601750

[28] Pompeo E, Mineo D, Rogliani P, Sabato AF, Mineo TC (2004) Feasibility and results of awake thoracoscopic resection of solitary pulmonary nodules. Ann Thorac Surg 78: 1761–1768. 10.1016/j.athoracsur.2004.05.083 15511470

[29] Pompeo E, Tacconi F, Mineo D, Mineo TC (2007) The role of awake video-assisted thoracoscopic surgery in spontaneous pneumothorax. J Thorac Cardiovasc Surg 133: 786–790. 10.1016/j.jtcvs.2006.11.001 17320585

[30] Vanni G, Tacconi F, Sellitri F, Ambrogi V, Mineo TC, et al (2010) Impact of awake videothoracoscopic surgery on postoperative lymphocyte responses. Ann Thorac Surg 90: 973–978. 10.1016/j.athoracsur.2010.04.070 20732526

[31] Cui F, Liu J, Li S, Yin W, Xin X, et al (2016) Tubeless video-assisted thoracoscopic surgery (VATS) under nonintubated, intravenous anesthesia with spontaneous ventilation and no placement of chest tube postoperatively. Journal of Thoracic Disease 8: 2226–2232. 10.21037/jtd.2016.08.02 27621880 PMC4999680

[32] Pompeo E, Tacconi F, Mineo TC (2011) Comparative results of non-resectional lung volume reduction performed by awake or non-awake anesthesia. European Journal of Cardio-thoracic Surgery 39: e51–e58. 10.1016/j.ejcts.2010.11.071 21397783

[33] Tacconi F, Pompeo E, Sellitri F, Mineo TC (2010) Surgical stress hormones response is reduced after awake videothoracoscopy. Interact Cardiovasc Thorac Surg 10: 666–671. 10.1510/icvts.2009.224139 20179134

[34] AlGhamdi ZM, Lynhiavu L, Moon YK, Moon MH, Ahn S, et al (2018) Comparison of non-intubated versus intubated video-assisted thoracoscopic lobectomy for lung cancer. J Thorac Dis 10: 4236–4243. 10.21037/jtd.2018.06.163 30174869 PMC6105965

[35] Guo Z, Yin W, Zhang X, Xu X, Liu H, et al (2016) Primary spontaneous pneumothorax: simultaneous treatment by bilateral non-intubated videothoracoscopy. Interact Cardiovasc Thorac Surg 23: 196–201. 10.1093/icvts/ivw123 27165732

[36] Hsiao CH, Chen KC, Chen JS (2017) Modified single-port non-intubated video-assisted thoracoscopic decortication in high-risk parapneumonic empyema patients. Surg Endosc 31: 1719–1727. 10.1007/s00464-016-5164-7 27519590

[37] Irons JF, Miles LF, Joshi KR, Klein AA, Scarci M, et al (2017) Intubated Versus Nonintubated General Anesthesia for Video-Assisted Thoracoscopic Surgery-A Case-Control Study. J Cardiothorac Vasc Anesth 31: 411–417. 10.1053/j.jvca.2016.07.003 27692903

[38] Jeon CS, Yoon DW, Moon SM, Shin S, Cho JH, et al (2018) Non-intubated video-assisted thoracoscopic lung biopsy for interstitial lung disease: a single-center experience. J Thorac Dis 10: 3262–3268. 10.21037/jtd.2018.05.144 30069322 PMC6051856

[39] Lan L, Cen Y, Zhang C, Qiu Y, Ouyang B (2018) A Propensity Score-Matched Analysis for Non-Intubated Thoracic Surgery. Med Sci Monit 24: 8081–8087. 10.12659/MSM.910605 30415268 PMC6410560

[40] McDonald CM, Pierre C, de Perrot M, Darling G, Cypel M, et al (2018) Efficacy and Cost of Awake Thoracoscopy and Video-Assisted Thoracoscopic Surgery in the Undiagnosed Pleural Effusion. Ann Thorac Surg 106: 361–367. 10.1016/j.athoracsur.2018.02.044 29577922

[41] Mineo TC, Pompeo E, Mineo D, Tacconi F, Marino M, et al (2006) Awake nonresectional lung volume reduction surgery. Ann Surg 243: 131–136. 10.1097/01.sla.0000182917.39534.2c 16371748 PMC1449981

[42] Mineo TC, Sellitri F, Tacconi F, Ambrogi V (2014) Quality of life and outcomes after nonintubated versus intubated video-thoracoscopic pleurodesis for malignant pleural effusion: comparison by a case-matched study. J Palliat Med 17: 761–768. 10.1089/jpm.2013.0617 24773212

[43] Noda M, Okada Y, Maeda S, Sado T, Sakurada A, et al (2012) Is there a benefit of awake thoracoscopic surgery in patients with secondary spontaneous pneumothorax? J Thorac Cardiovasc Surg 143: 613–616. 10.1016/j.jtcvs.2011.07.067 22104684

[44] Tacconi F, Pompeo E, Fabbi E, Mineo TC (2010) Awake video-assisted pleural decortication for empyema thoracis. Eur J Cardiothorac Surg 37: 594–601. 10.1016/j.ejcts.2009.08.003 19762250

[45] Tacconi F, Pompeo E, Mineo TC (2009) Duration of air leak is reduced after awake nonresectional lung volume reduction surgery. European Journal of Cardio-thoracic Surgery 35: 822–828. 10.1016/j.ejcts.2009.01.010 19233672

[46] Liang H, Liu J, Wu S, Zhang Y, Liu H, et al (2019) Non-intubated spontaneous ventilation offers better short term outcome for mediastinal tumor surgery. Ann Thorac Surg. 10.1016/j.athoracsur.2019.04.052 31181206

[47] Tonnesen E, Hohndorf K, Lerbjerg G, Christensen NJ, Huttel MS, et al (1993) Immunological and hormonal responses to lung surgery during one-lung ventilation. Eur J Anaesthesiol 10: 189–195. 8495681

[48] De Conno E, Steurer MP, Wittlinger M, Zalunardo MP, Weder W, et al (2009) Anesthetic-induced improvement of the inflammatory response to one-lung ventilation. Anesthesiology 110: 1316–1326. 10.1097/ALN.0b013e3181a10731 19417610

[49] Kozian A, Schilling T, Freden F, Maripuu E, Rocken C, et al (2008) One-lung ventilation induces hyperperfusion and alveolar damage in the ventilated lung: an experimental study. Br J Anaesth 100: 549–559. 10.1093/bja/aen021 18308740

[50] Howard-Quijano K, Takamiya T, Dale EA, Yamakawa K, Zhou W, et al (2017) Effect of Thoracic Epidural Anesthesia on Ventricular Excitability in a Porcine Model. Anesthesiology 126: 1096–1106. 10.1097/ALN.0000000000001613 28358748

[51] Vollmer C, Nommensen J, Watolla M, Bauer I, Picker O (2016) Influence of thoracic epidural anesthesia on gastric oxygenation during hypothermia and hemorrhage. Auton Neurosci 195: 1–7. 10.1016/j.autneu.2016.01.004 26905213

[52] Trejo Bittar HE, Doberer D, Mehrad M, Strollo DC, Leader JK, et al (2017) Histologic Findings of Severe/Therapy-Resistant Asthma From Video-assisted Thoracoscopic Surgery Biopsies. Am J Surg Pathol 41: 182–188. 10.1097/PAS.0000000000000777 28079597 PMC5234856

[53] Matsuoka K, Kuroda A, Kang A, Imanishi N, Nagai S, et al (2013) Surgical results of video-assisted thoracic surgery and risk factors for prolonged hospitalization for secondary pneumothorax in elderly patients. Ann Thorac Cardiovasc Surg 19: 18–23. 10.5761/atcs.oa.12.01909 22971717

[54] Papadima A, Boutsikou M, Lagoudianakis EE, Kataki A, Konstadoulakis M, et al (2009) Lymphocyte apoptosis after major abdominal surgery is not influenced by anesthetic technique: a comparative study of general anesthesia versus combined general and epidural analgesia. J Clin Anesth 21: 414–421. 10.1016/j.jclinane.2008.10.015 19833274

[55] Velcic Brumnjak S, Rakovac I, Papez Kinkela D, Bukal K, Sestan B, et al (2018) Postoperative Regional Analgesia Is Effective in Preserving Perforin-Expressing Lymphocytes in Patients After Total Knee Replacement. Med Sci Monit 24: 5320–5328. 10.12659/MSM.909385 30063033 PMC6083937

[56] Zhang K, Chen HG, Wu WB, Li XJ, Wu YH, et al (2019) Non-intubated video-assisted thoracoscopic surgery vs. intubated video-assisted thoracoscopic surgery for thoracic disease: a systematic review and meta-analysis of 1,684 cases. J Thorac Dis 11: 3556–3568. 10.21037/jtd.2019.07.48 31559062 PMC6753434

