# Supplementary material for: Non-intubated anesthesia in patients undergoing video-assisted thoracoscopic surgery: A systematic review and meta-analysis
Source: PLoS One. 2019 Nov 12;14(11):e0224737. doi: 10.1371/journal.pone.0224737 (PMC6850529; doi:10.1371/journal.pone.0224737)
Supplement: S1 File — (DOCX) [file pone.0224737.s001.docx]

Pubmed

1. (Thoracic surgery, Video-Assisted)[Mesh] or Surgeries, Video-Assisted Thoracic [af] or Surgery, Video-Assisted Thoracic [af] or Thoracic Surgeries, Video-Assisted [af] or Thoracic surgery, Video-Assisted [af] or Video-Assisted Thoracic Surgeries [af] or Video-Assisted Thoracic Surgery [af] or Surgeries, Video-Assisted Thoracoscopic [af] or Surgery, Video-Assisted Thoracoscopic [af] or Thoracoscopic Surgeries, Video-Assisted [af] or Thoracoscopic Surgery, Video-Assisted [af] or Video Assisted Thoracoscopic Surgery [af] or Video Assisted Thoracoscopic Surgeries [af] or Video-Assisted Thoracic Surgery [af] or Video Assisted Thoracic Surgery [af] or Surgery, Thoracic, Video-Assisted [af] or VATS [af] or VATSs[af].
2. Non-intubated anesthesia [af] or non-intubated [af] or nontracheal intubation [af] or epidural anesthesia [af] or thoracic epidural [af] or paravertebral block [af] or intercostal block [af] or local anesthesia [af] or regional anesthesia [af] or tubeless [af]or awake [af] or sedation [af]
3. 1 and 2

Cochrane

1. (Thoracic surgery, Video-Assisted)[Mesh] or Surgeries, Video-Assisted Thoracic [at] or Surgery, Video-Assisted Thoracic [at] or Thoracic Surgeries, Video-Assisted [at] or Thoracic surgery, Video-Assisted [at] or Video-Assisted Thoracic Surgeries [at] or Video-Assisted Thoracic Surgery [at] or Surgeries, Video-Assisted Thoracoscopic [at] or Surgery, Video-Assisted Thoracoscopic [at] or Thoracoscopic Surgeries, Video-Assisted [at] or Thoracoscopic Surgery, Video-Assisted [at] or Video Assisted Thoracoscopic Surgery [at] or Video Assisted Thoracoscopic Surgeries [at] or Video-Assisted Thoracic Surgery [at] or Video Assisted Thoracic Surgery [at] or Surgery, Thoracic, Video-Assisted [at]
2. Non-intubated anesthesia [at] or non-intubated [at] or nontracheal intubation [at] or epidural anesthesia [at] or thoracic epidural [at] or paravertebral block [at] or intercostal block [at] or local anesthesia [at] or regional anesthesia [at] or tubeless [at]or awake [at] or sedation [at]
3. 1 and 2

Embase

1. “video assisted thoracoscopic surgery”/exp OR” thoracic surgery, video-assisted” OR “VATS” OR “video assisted thoracic surgery” OR “videothoracoscopic surgery”
2. “non-intubated anesthesia” OR “non-intubated” OR “nontracheal intubation” OR “epidural anesthesia” OR “thoracic epidural” OR “paravertebral block” OR “intercostal block” OR “local anesthesia” OR “regional anesthesia” OR “tubeless” OR “awake” OR “sedation”
3. #1 AND #2
