# Supplementary material for: Non-intubated anesthesia in patients undergoing video-assisted thoracoscopic surgery: A systematic review and meta-analysis
Source: PLoS One. 2019 Nov 12;14(11):e0224737. doi: 10.1371/journal.pone.0224737 (PMC6850529; doi:10.1371/journal.pone.0224737)
Supplement: S1 Table — (DOC) [file pone.0224737.s003.doc]

***Supplementary Table 1 Characteristics of included studies [ordered by study ID]***

| First Author | Year | Country | Study design | Sample size | Diseases | Type of surgery | | Type of NIA |
| --- | --- | --- | --- | --- | --- | --- | --- | --- |
| No | 2018 | Korea | Retrospective study | 30/30 | Lung cancer | | Lobectomy | INB and vagal nerve block combined with infusion of propofol (2-4μg/ml) and dexmedetomidine (0.6-0.8μg/kg/min) |
| Ambrogi | 2017 | Italy | Retrospective study | 48/13 | Lung colorectal oligometastases | Metastasectomy | | INB combined with infusion of propofol (0.5 mg/kg) and remifentanil (15 μg/kg/min) |
| Chen | 2011 | China | Observational comparative study | 30/30 | Stage I or II NSCLC | Lobectomy | | TEA combined with TCI infusion of propofol (10 mg/ml) |
| Cui | 2016 | China | Observational comparative study | 89/82 | Pulmonary bulla, mediastinal tumor, etc | Sympathectomy, bullectomy, mediastinal tumor resection | | TEA combined with TCI of propofol (2.0-3.0 μg/ml) and sulfentanil (0.1-0.2 μg/kg) |
| Guo #1 | 2016 | China | Retrospective study | 48/92 | Lung tumor | Anatomic segmentectomy | | TEA combined with TCI of propofol (2.0-3.0 μg/ml) and sulfentanil (0.1-0.2 μg/kg) |
| Guo #2 | 2016 | China | Retrospective study | 15/22 | Primary spontaneous pneumothorax | Bullectomy | | TEA combined with TCI of propofol (2.0-3.0 μg/ml) and sulfentanil (0.1-0.2 μg/kg) |
| Hsiao | 2017 | China | Retrospective study | 12/21 | Parapneumonic empyema | VAFTS decortication | | Skin and intercostal space block by 5 ml of 0.5% bupivacaine hydrochloride (5mg/ml) |
| Irons | 2017 | United Kingdom | Retrospective, case-control study | 31/31 | Not specified | Bullectomy, biopsy, isolated pleurodesis | | LMA Classic or the i-gel supraglottic airway combined with propofol (1.0-2.0 mg/kg) and sevoflurane at 0.8-1.0 MAC. |
| Jeon | 2018 | Korea | Retrospective study | 10/25 | Not specified | Lung biopsy | | TEA combined with remifentanil (0.0-0.5μg/kg/min) and dexmdetomidine (0.3-0.5μg/kg/hr) |
| Lan | 2018 | China | Retrospective, case-control study | 119/119 | Not specified | Lobectomy | | TEA and INB combined with remifentanil and propofol |
| Liu | 2015 | China | Randomized control study | 167/180 | Not specified | Pulmonary wedge resection, bullectomy, lobectomy | | TEA combined with TCI of propofol (2.0-3.0 μg/ml) and sulfentanil (0.1-0.2 μg/kg) |
| Liu | 2016 | China | Retrospective, propensity score matching study | 136/136 | NSCLC | Lobectomy or segmentectomy | | TEA and INB combined with TCI of propofol (2.0-3.0 μg/ml) and sulfentanil (0.1-0.2 μg/kg) |
| McDonald | 2018 | Canada | Retrospective study | 78/99 | Exudative pleural effusions | Pleural biopsy | | Olympus LTF-160 semi-rigid pleuroscope, 10mm flexible port and sedation |
| Mineo | 2006 | Italy | Retrospective study | 12/12 | Pulmonary emphysema | Intentional unilateral LVRS | | TEA |
| Mineo | 2014 | Italy | Retrospective, case-matched study | 231/231 | Malignant pleural effusion | Talc pleurodesi | | INB and intravenous administration of remifentanil (15 mcg/kg/min) |
| Mineo | 2017 | Italy | Retrospective study | 55/13 | Pulmonary oligometastases | Metastasectomy | | INB and intravenous administration of midazolam 0.03-0.1 mg/kg, propofol 0.5 mg/kg and remifentanil (15 mcg/kg/min) |
| Nezu | 1997 | Japan | Retrospective study | 32/38 | Spontaneous pneumothorax | Pulmonary wedge resection | | INB, pleural local anesthesia and iv administration of butorphanol 1 mg, diazepam 5 mg |
| Noda | 2012 | Japan | Retrospective study | 57/42 | Secondary spontaneous pneumothorax | Pulmonary wedge resection | | TEA and local anesthesia |
| Pompeo | 2004 | Italy | Randomized control study | 30/30 | Pulmonary nodule | Pulmonary wedge resection | | TEA |
| Pompeo | 2007 | Italy | Randomized control study | 21/22 | Primary spontaneous pneumothorax | Bullectomy | | TEA |
| Pompeo | 2011 | Italy | Observational, prospective investigation | 41/19 | Pulmonary emphysema | Unilateral non-resectional LVRS | | TEA |
| Pompeo | 2012 | Italy | Randomized control study | 32/31 | Pulmonary emphysema | Unilateral non-resectional LVRS | | TEA |
| Pompeo | 2013 | Italy | Randomized control study | 20/20 | Malignant pleural effusion | Talc pleurodesis | | TEA |
| Tacconi | 2009 | Italy | Retrospective study | 66/66 | Pulmonary emphysema | Resectional LVRS | | TEA |
| Tacconi #1 | 2010 | Italy | Retrospective study | 19/19 | Empyema thoracis | Pleural decortication | | TEA or paravertebral blocks |
| Tacconi #2 | 2010 | Italy | Perspective, pilot investigation | 11/10 | Non-oncological thoracic disease | VATS | | TEA |
| Vanni | 2010 | Italy | Prospective randomized two-arm study | 25/25 | Nonmalignant pulmonary conditions | Talc pleurodesis | | TEA |

**Comments:** NIA, nonintubated anesthesia; INB, intercostal nerve block; NSCLC, non-small-cell lung cancer; TEA, thoracic epidural anesthesia; TCI, target controlled infusion; VAFTS, video-assisted flexible thoracoscopic surgery; LMA, laryngeal mask airway; MAC, minimum alveolar concentration; LVRS, lung volume reduction surgery; VATS, video-assisted thoracoscopic surgery.
